# Editorial: The Role of Glycans in Infectious Disease

**DOI:** 10.3389/fmicb.2022.921436

**Published:** 2022-05-12

**Authors:** Iván Martínez-Duncker, Fabrizio Chiodo, Héctor M. Mora-Montes, Gerardo R. Vasta

**Affiliations:** ^1^Laboratorio de Glicobiología Humana y Diagnóstico Molecular, Centro de Investigación en Dinámica Celular, Instituto de Investigación en Ciencias Básicas y Aplicadas, Universidad Autónoma del Estado de Morelos, Cuernavaca, Mexico; ^2^Department of Molecular Cell Biology and Immunology, Amsterdam UMC, Vrije Universiteit Amsterdam, Amsterdam, Netherlands; ^3^Institute of Biomolecular Chemistry, National Research Council (CNR) Pozzuoli Napoli, Pozzuoli Napoli, Italy; ^4^División de Ciencias Naturales y Exactas, Departamento de Biología, Universidad de Guanajuato, Guanajuato, Mexico; ^5^Department of Microbiology and Immunology, University of Maryland School of Medicine, UMB and Institute of Marine and Environmental Technology, University of Maryland, Baltimore, MD, United States

**Keywords:** tuberculosis, Chagas, toxoplasmosis, galectins, *Sporothrix*, fungal, HIV, COVID

Virtually all eukaryotic and bacterial cells, as well as many enveloped viruses, display carbohydrates of variable complexity associated to their macromolecules (e.g., glycoproteins, glycolipids), as well as polysaccharides. The carbohydrate moieties of both soluble and cell-associated glycoconjugates encode complex information that is “decoded” by specific carbohydrate–binding proteins. These protein–carbohydrate interactions are ubiquitous and essential to all biological systems.

In metazoans, for example, recognition of endogenous (“self”) glycans, either soluble or displayed on the cell surface, is critical not only for specific interactions between cells that facilitate cell adhesion and migration, but as the initiator of a functional crosstalk that modulates cell homeostatic balance, including the regulation of both innate and adaptive immune functions. In contrast, recognition of exogenous (“non-self”) carbohydrates on the surface of viruses, microbes, and parasites by the host's glycan-binding proteins frequently constitutes the first step in the innate immune response to infectious challenge.

As microbes, parasites and some viruses are also endowed of a diverse repertory of adhesins, lectins, hemagglutinins, and other glycan-binding proteins that facilitate their adhesion and host entry, the outcome of this interplay of reciprocal glycan recognition may result in either infectious disease, a successful host immune response, or the establishment of a mutualistic association, such as the commensal microbiota.

Volume 1 of “*The Role of Glycans in Infectious Disease*” Research Topic offers a series of high-quality articles that share an ample view of glycan host-pathogen interactions involving bacterial, fungal, parasitic, and viral infections, as well as the different approaches used to study them and to develop diagnostic and therapeutic applications.

A wide number of glycoproteins across different bacterial species are involved in pathogenicity and virulence. Tuberculosis, caused by infection with *Mycobacterium tuberculosis* (Mtb), is a communicable disease that is a major cause of ill health and one of the leading causes of death worldwide. Until the coronavirus (COVID-19) pandemic, tuberculosis was the leading cause of death from a single infectious agent (World Health Organization, 2021a). It is known that Mtb expresses a wide range of *O*-mannosylated proteins involved in the pathogenesis and immune response to tuberculosis. In an original research article, Jia et al. highlight this role by using *Mycobacterium smegmatis* as a study model, demonstrating that the mycobacterium protein *O*-mannosyltransferase, that catalyzes the initial step of protein *O*-mannosylation, is required for growth and resistance to lysozyme and acidic stress, determining survival within macrophages, as well as in modulating the host innate immune responses.

Endogenous lectins such as galectins, a large family of carbohydrate binding proteins with members in nearly every lineage of multicellular life, are known to play important roles in modulating host cell function, but also in the binding of non-self glycans on the surface of potentially pathogenic microorganisms, mediating recognition and effector functions in innate immunity (Verkerke et al., 2022). In an original research article, Wu et al. present data regarding the binding specificities of galectin-3 (Gal-3), the first galectin shown to engage bacterial glycans, as well as its C-terminal domain, indicating that differently from other galectins, such as Gal-8, the C-terminal domain of Gal-3 is not sufficient to kill bacteria, and that the N-terminal is required for both high-affinity microbial glycan interactions and the ability to kill microbes.

Antimicrobial resistance is a global threat to the very core of modern medicine and the sustainability of an effective, global public health response to the enduring threat from infectious diseases (Regional Office for South-East Asia WHO, 2016). Since bacterial glycosylation is different from human glycosylation, these metabolic pathways constitute promising antibacterial targets. In a timely review, Yakovlieva et al. describe the current status and promise for the future of using bacterial glycosylation to develop novel antibacterial strategies by focusing on unique glycosylation systems in bacterial pathogens and their role in bacterial homeostasis and infection, with an emphasis on virulence factors and highlighting recent advances to inhibit the enzymes involved in these glycosylation systems.

Fungal diseases have been continually neglected over the years (Rodrigues and Nosanchuk, 2020) and understanding the immune response to fungal infection is key in unraveling their pathogenesis. The fungal cell wall is a robust and dynamic structure that protects the cell from the changes in the extracellular environment, but that also is the immediate contact point with host cells, containing antigenic determinants, glycoproteins involved in the adhesion to host tissues, and most of the pathogen-associated molecular patterns that are recognized by the host immune system. In an original research article, Villalobos-Duno et al. share a comparative study on the cell wall glycosylation of *Sporothrix schenckii* and *Sporothrix brasiliensis* strains, the main causative agents of sporotrichosis, a human subcutaneous mycosis. Important differences regarding rhamnose-to-β-glucan ratio and structural differences in rhamnomannan were observed and the authors associated changes to the different virulence degrees of the studied strains, interestingly expressing them through a linear equation.

Infections caused by parasitic protozoans and helminths are among the world's leading causes of death, including malaria, one of the leading global health burdens, estimated in 2020 to have caused 241 million cases of malaria worldwide and an estimated number of 627 000 deaths (World Health Organization, 2021b). In a detailed review, Goerdeler et al. describe the role of glycans and lectins in the pathogenesis and host defense mechanisms of plasmodium parasites that cause malaria, explaining the basics of the pathogen glycosylation pathways and the host glycans that participate in the disease, sharing their perspectives on intervention sites for malaria therapy, including vaccine development and glycan-based drug targets. Also in an original research article, Ricci-Azevedo et al. reveal a mechanism by which *Toxoplasma gondii* lectin type microneme proteins inhibit the host inflammatory response to favor its success in the early stage of toxoplasmosis, a widely distributed parasitic zoonotic infection of importance to public health and animal production (de Barros et al., 2022). Chagas disease is a neglected tropical disease caused by infection with the parasite *Trypanosoma cruzi*, endemic to Latin America, but found in immigrant populations worldwide (Álvarez-Hernández et al., 2021). In a focused review, Poncini et al. discuss the role of galectin-driven circuits in modulating both *T. cruzi* infection and immunoregulation, clearly articulating the decisive roles that galectins play during the life cycle of *T. cruzi*.

The COVID-19 pandemic and the study of the pathogenic mechanisms involving SARS-CoV-2 infection have highlighted the importance of glycans as key participants in the interphase of virus-host interactions. An interesting aspect shared in an original research article by Breiman et al. shed light into the potential protective role against COVID-19 infection of naturally occurring antibodies against common glycan epitopes. COVID-19 patients were found to present lower levels of anti-Tn antibodies than controls, pointing to the potential protective role of these antibodies. This finding contributes to the ongoing and complex discussion regarding differences in susceptibility amongst the human population and if boosting this natural protection through anti-glycan antibody epitopes could be considered a prophylactic therapy. Also, in an original research article, Schwedler et al. report on the *N*-glycosylation of total IgG1, total IgG2, and anti-Spike IgG1 isolated from plasma of severe COVID-19 patients by means of MALDI-TOF-MS, showing that anti-Spike IgG1 fucosylation and galactosylation had the strongest variation during the disease course.

Another viral pathogen included in this Research Topic is the Human Immunodeficiency Virus (HIV), well-known to use glycans and host lectins at different stages of its life cycle. In a focused review, Segura et al. discuss the pathogenic mechanisms involving the interaction between HIV-1 envelope glycans and their binding to L-selectin on CD4+ T lymphocytes to facilitate viral adhesion and entry and the role of L-selectin shedding in viral release, suggesting that the regulation of L-selectin is a promising target for developing anti-HIV therapies.

A physiological perspective regarding the study of the glycan host-pathogen interactions offers a better understanding of the processes involving different types of cells and tissues that come into place during infection. In a detailed mini-review, Argüeso et al. describe the ocular glycocalyx barrier that occurs in the interface between the ocular surface epithelia and the external environment and its role in the pathogenesis of bacterial, viral, fungal and parasitic infection. Also, in a dedicated review, Jung and Kim describe the intestinal models for studying normal and disease host-microbiome interactions and pathways, including the current state of the art for *in vitro* cell-based models of the small intestine system to replace animal models, including *ex vivo*, 2D culture, organoid, lab-on-a-chip, and 3D culture models.

An important benefit of understanding the role of glycans and glycan-binding proteins in host-pathogen interactions is to develop novel diagnostic tools and therapeutics that can positively impact prompt diagnosis and better outcomes. McKitrick et al. present a very interesting perspective, reporting on a workshop organized jointly by the National Institute of Allergy and Infectious Diseases and the National Institute of Dental and Craniofacial Research that addressed the use of emerging glycoscience tools and resources to advance the investigation of glycans and their roles in microbe-host interactions, immune-mediated diseases, and immune cell recognition and function.

Although most contributions involved human pathogens, we had the opportunity to include an extensive review by Villa-Rivera et al. that describes the beneficial plant-microbe interactions and defense mechanisms established by arabinogalactans and arabinogalactan proteins found in the plant cell wall or plasma membrane, including the interplay with pathogenic fungal and bacterial enzymes that degrade them to establish infections and that result in plant defense responses.

This Research Topic underscores the diverse and expanding role of glycans and glycan-binding proteins in different infectious diseases, presenting it as a promising field to discover novel mechanisms involved in host-pathogen interactions, that harbor the potential for improved design of novel diagnostic tools and therapeutics against infectious diseases.

## Author Contributions

All authors listed have made a substantial, direct, and intellectual contribution to the work and approved it for publication.

## Funding

GV was supported by grants IOS-1656720 from the National Science Foundation, and grant R01 GM070589 from the National Institutes of Health. HM-M was supported by grants from the Consejo Nacional de Ciencia y Tecnología FC2015-02-834; Ciencia de Frontera 2019-6380 and Red Temática Glicociencia en Salud.

## Conflict of Interest

The authors declare that the research was conducted in the absence of any commercial or financial relationships that could be construed as a potential conflict of interest.

## Publisher's Note

All claims expressed in this article are solely those of the authors and do not necessarily represent those of their affiliated organizations, or those of the publisher, the editors and the reviewers. Any product that may be evaluated in this article, or claim that may be made by its manufacturer, is not guaranteed or endorsed by the publisher.
